# Supplementary material for: River thorium concentrations can record bedrock fracture processes including some triggered by distant seismic events
Source: Nat Commun. 2023 Apr 26;14:2395. doi: 10.1038/s41467-023-37784-3 (PMC10133341; doi:10.1038/s41467-023-37784-3)
Supplement: Supplementary file 1 — Supplementary Information [file 41467_2023_37784_MOESM1_ESM.pdf]

River thorium concentrations can record bedrock fracture processes including some triggered by distant seismic events

Supplementary Figures and Tables

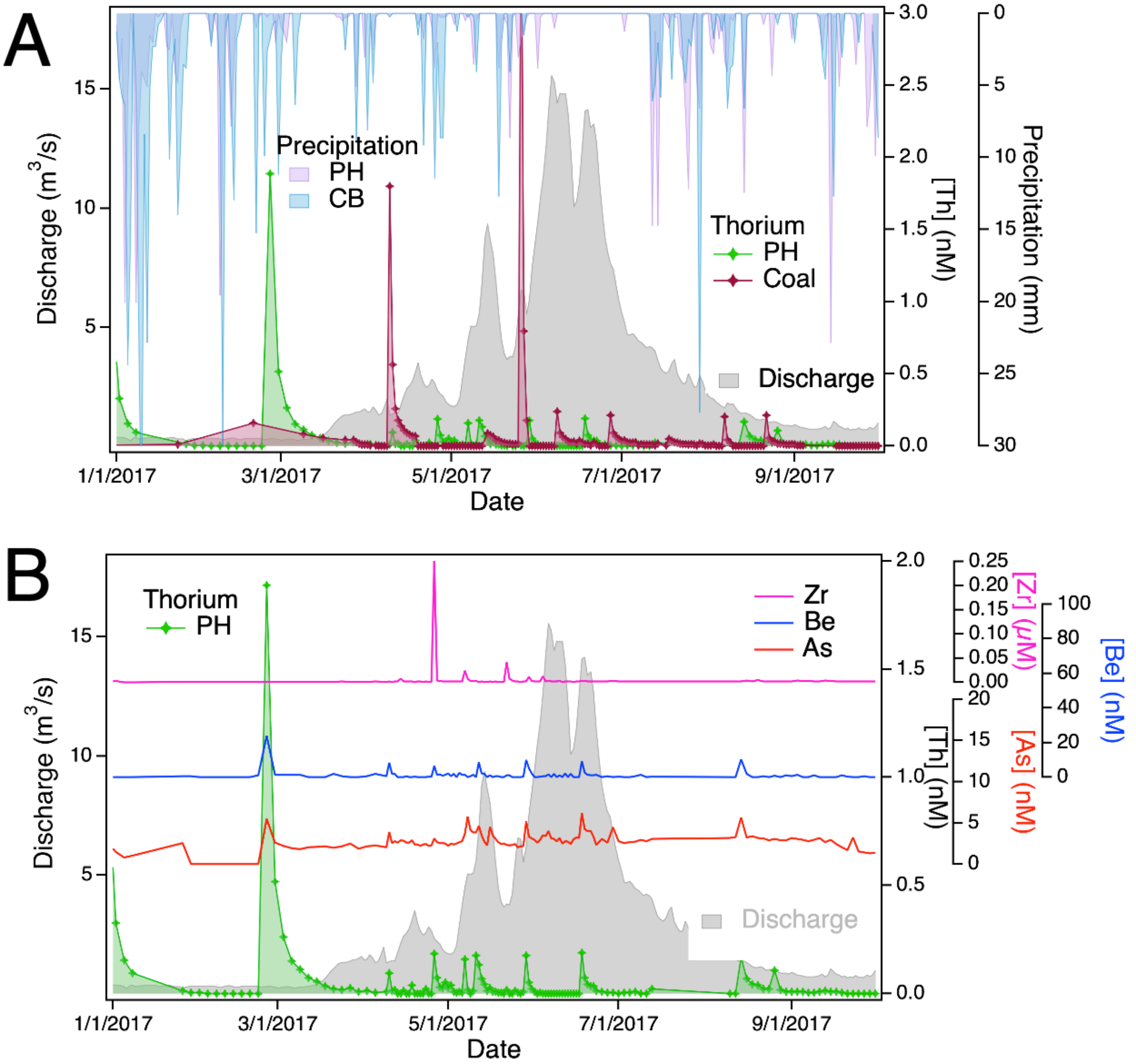

Figure S1 East River and Coal Creek data for 9 months of 2017 extending the data in Fig. 1. **A**) Comparison of East River Pump House (PH) and Coal Creek thorium data in calendar year 2016 with East River discharge and precipitation at PH and Crested Butte (CB). (**B**) Comparison of East River thorium concentrations with zinc, beryllium and arsenic.

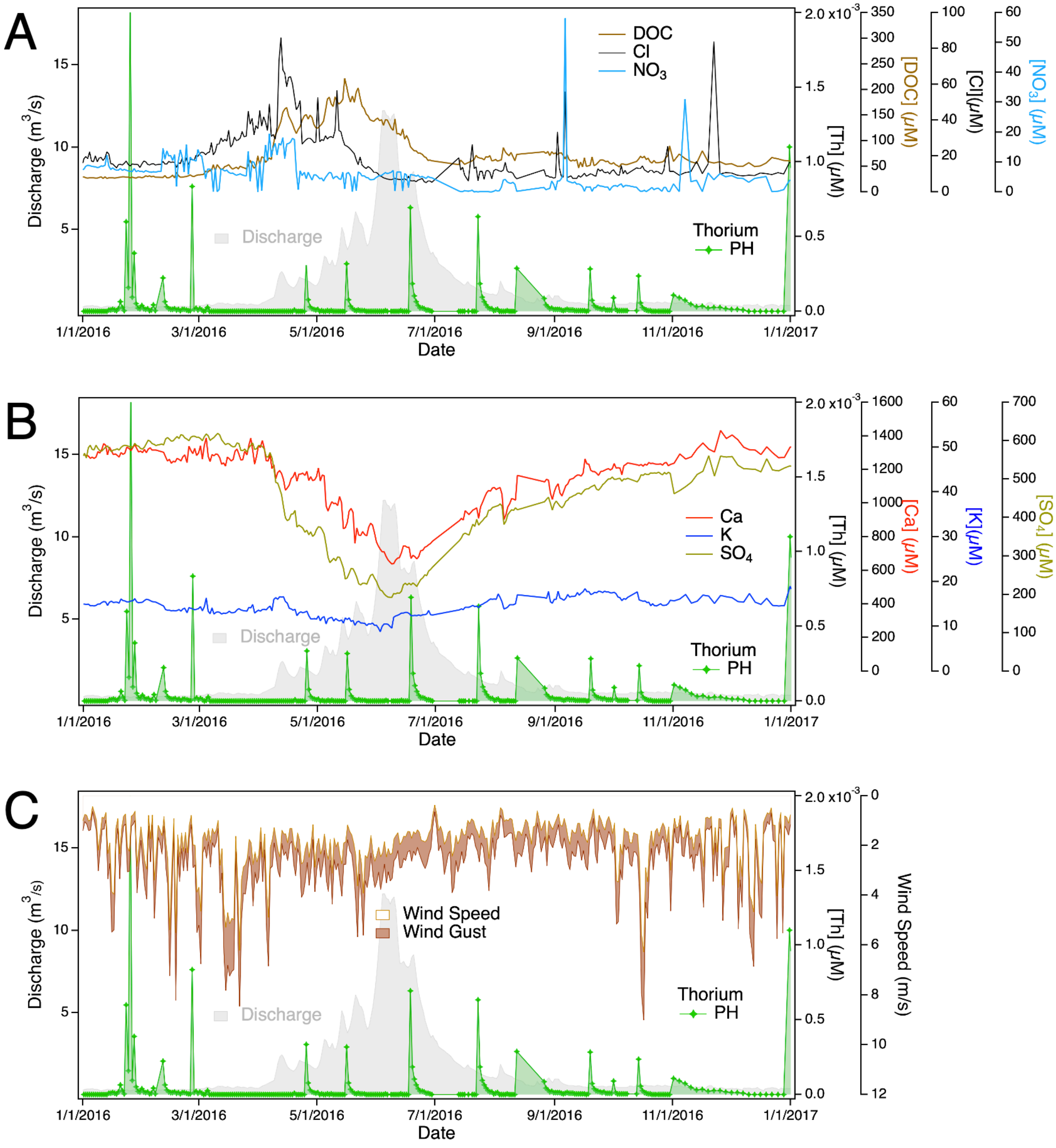

Figure S2 (partial) Comparison of East River thorium data with geochemical and meteorological data for calendar year 2017. (**A**) Dissolved organic carbon (DOC), chloride and nitrate are solutes that exhibit flushing behavior during snowmelt. (**B**) Calcium, potassium and sulfate are solutes released by shale bedrock weathering. (**C**) Wind speed and gust, which can cause tree sway and bedrock fracturing by roots.

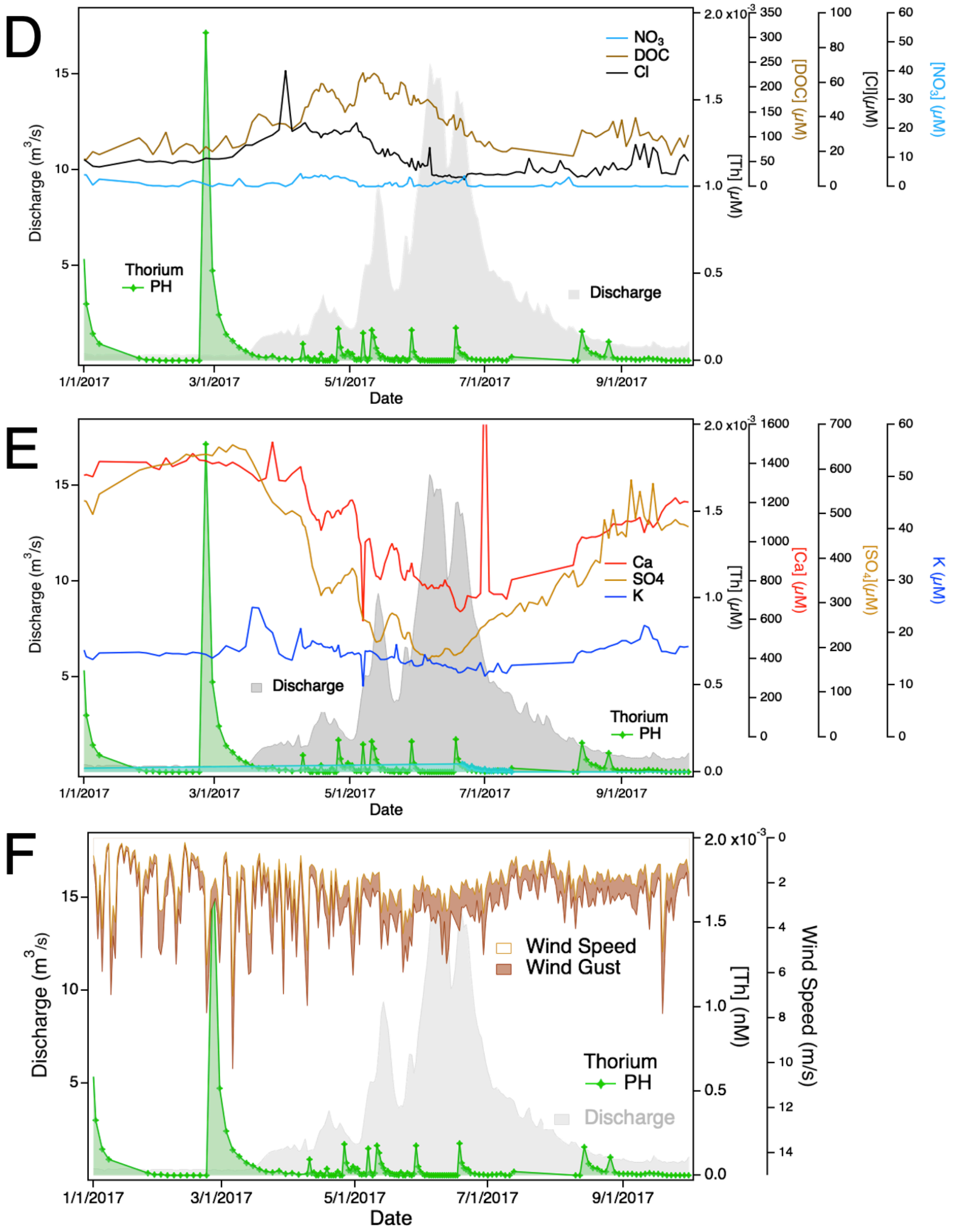

Figure S2 (continued) Comparison of East River thorium data with geochemical and meteorological data for 10 months in 2018. (**D**) Dissolved organic carbon (DOC), chloride and nitrate are solutes that exhibit flushing behavior during snowmelt. (**E**) Calcium, potassium and sulfate are solutes released by shale bedrock weathering. (**F**) Wind speed and gust, which can cause tree sway and bedrock fracturing by roots.

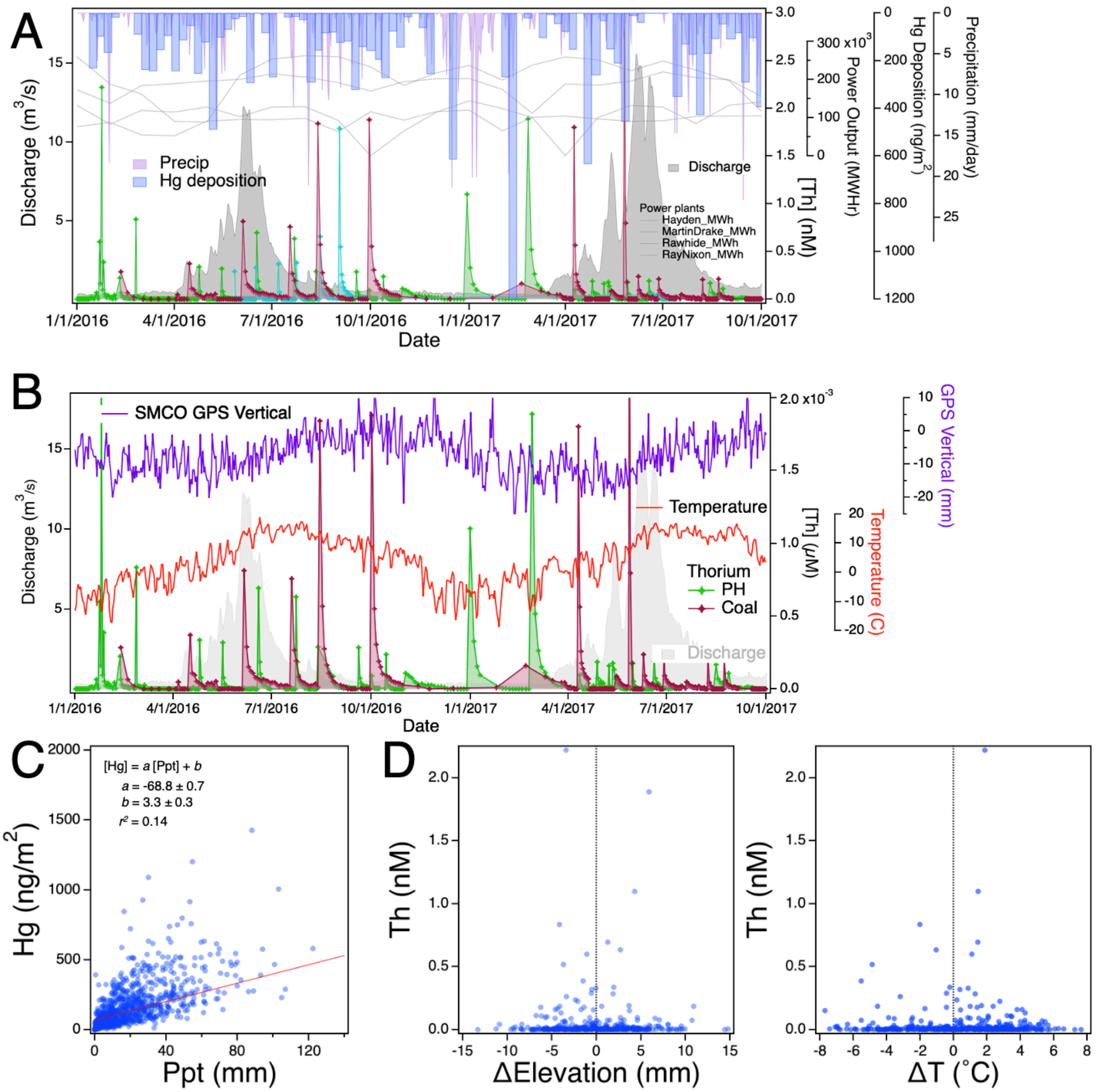

Figure S3 Comparison of East River and Coal Creek thorium data with mercury deposition and changes in ground surface elevation and temperature. (**A**) Mercury deposition at MDN Site CO97, Buffalo Pass - Summit Lake, CO, compared with East River precipitation from the KCOMKRET site. (**B**) Daily vertical elevation and temperature changes at Snowmass, CO. (**C**) Correlation plot of Hg deposition *versus* precipitation. (**D**) Correlation plots of Th *versus* detrended elevation and temperature.

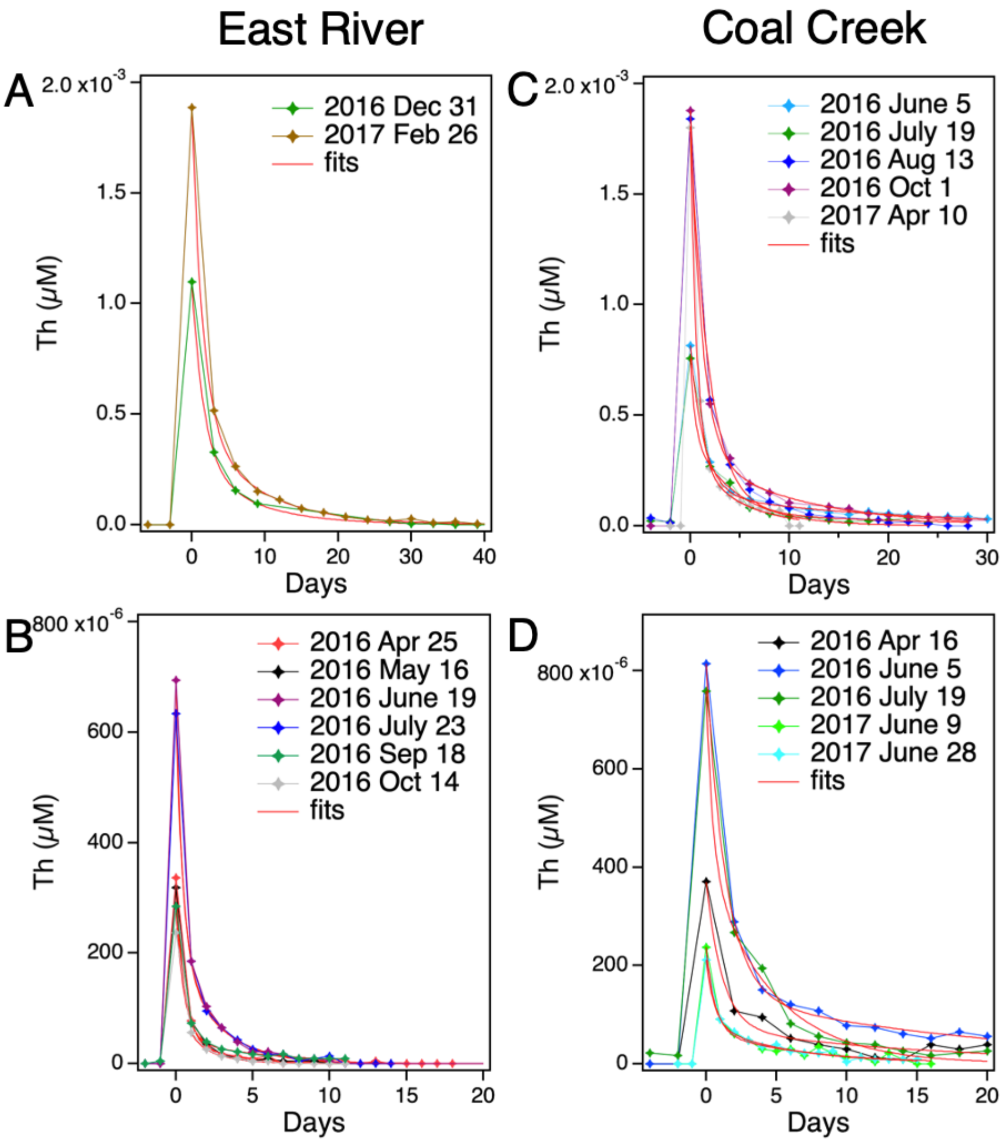

Figure S4 Biexponential fits to thorium excursions from Pump House location East River (A,B) and Coal-11 location Coal Creek (C,D). For each location that data are displayed in two plots of higher and lower concentrations for clarity of viewing. Best fit values are given in **Table S1**

Date	Site	[Th] μM	τ_1 (days)	τ_1 (days)	Notes
1/23/2016	PH	0.60	—	—	Non-exponential trend
1/25/2016	PH	2.2	—	—	Non-exponential trend
2/12/2016	CC	0.28	—	—	Missing data
2/26/2016	PH	0.84	1.5	8.2	
4/5/2016	CC	0.05	—	—	Too low concentration
4/16/2016	CC	0.38	1.2	16	
4/25/2016	PH	0.33	0.54	3.3	
5/16/2016	PH	0.31	0.52	2.4	
6/5/2016	CC	0.81	1.4	19	
6/18/2016	PH	0.69	0.38	2.3	
7/19/2016	CC	0.76	0.46	4.5	
7/23/2016	PH	0.63	0.48	2.6	
8/12/2016	PH	0.29	—	—	Missing data
8/13/2016	CC	1.80	1.9	18	
9/18/2016	PH	0.28	0.54	5.9	
10/1/2016	PH	0.09	—	—	
10/1/2016	CC	1.90	1.2	10.2	
10/14/2016	PH	0.24	0.56	1.8	
11/1/2016	PH	0.11			
12/31/2016	PH	1.10	1.6	7.8	
2/26/2017	PH	1.9	1.5	8.2	
4/10/2017	CC	1.8	0.63	4.5	
6/9/2017	CC	0.23	0.63	6.7	
6/28/2017	CC	0.21	0.60	5.8	
**Average**			**0.92 ± 0.5**	**7.5 ±5.5**	

Table S1 Results of biexponential fits to thorium excursion data from Fig. S5.

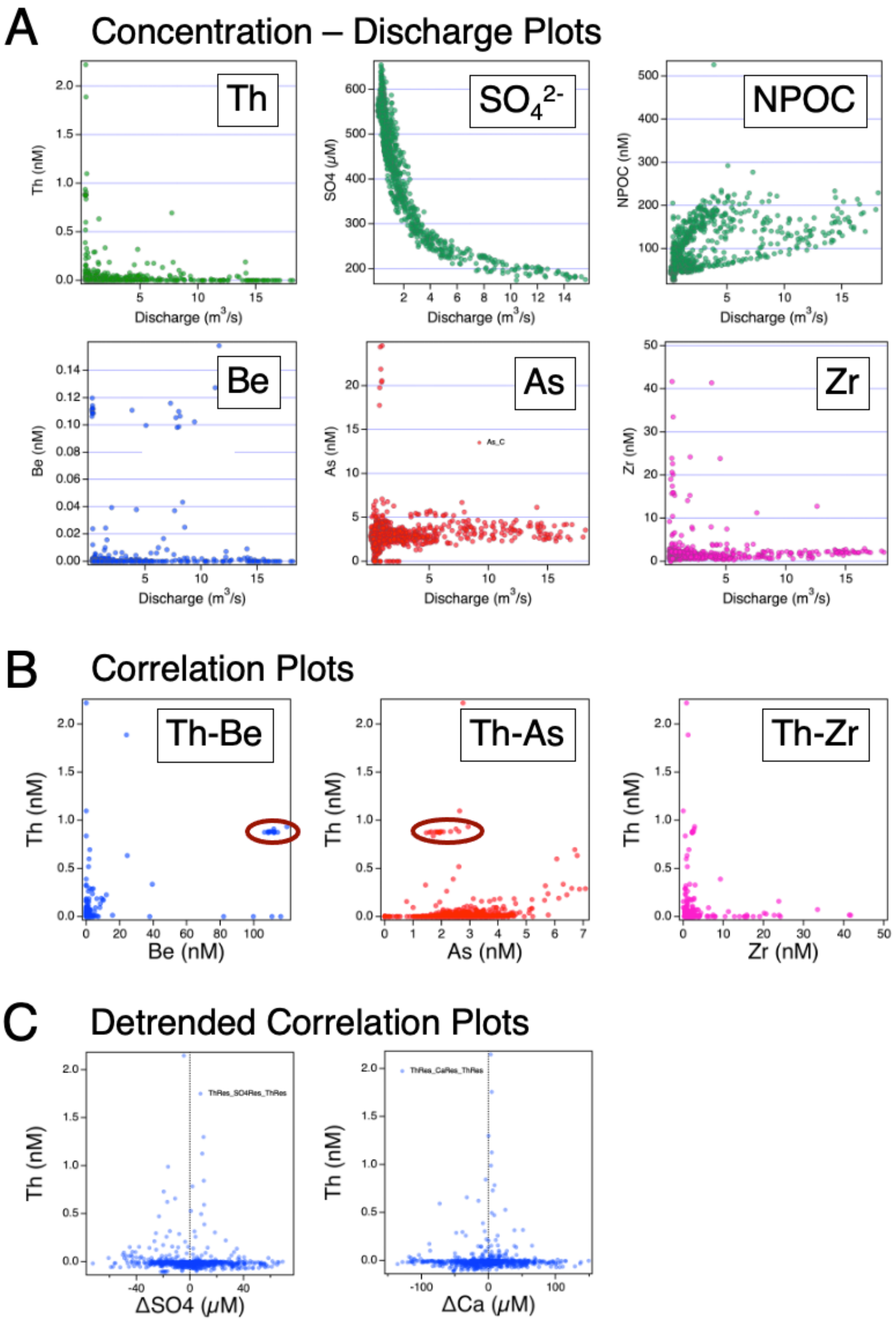

Figure S5 Solute trends in the East River (**A**) Concentration discharge plots. (**B**) Thorium – element correlation plots. The points highlighted by the ovals in Th-Be and Th-As are the days when abrupt increases in Be and As concentrations were associated with Th excursions. (**C**) Detrended thorium – element correlation plots.

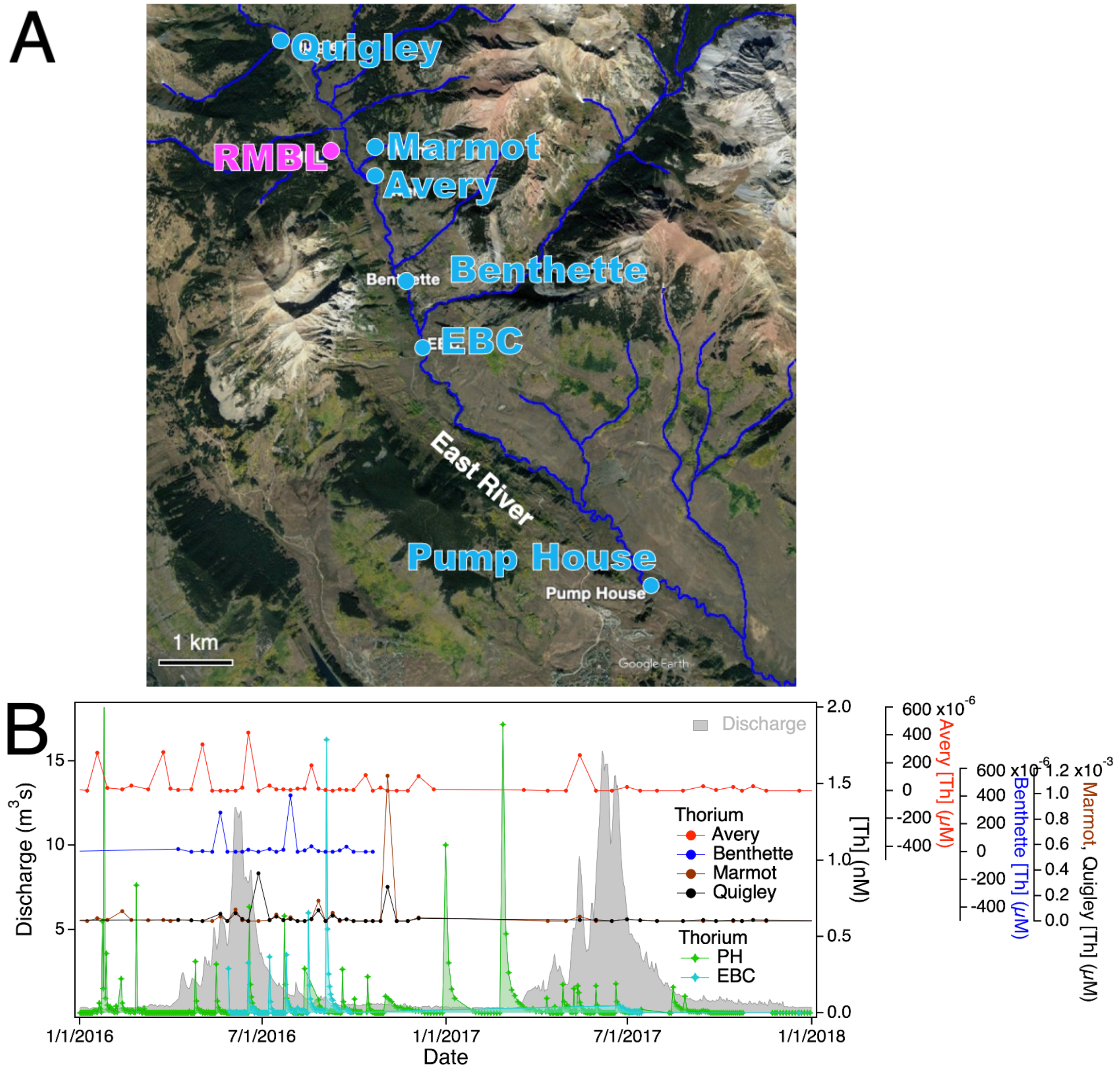

Figure S6 Thorium data from East River locations and tributaries upstream of the Pump House. (**A**) Sampling locations. Satellite image was obtained from Google Earth Pro. (**B**) Time-series thorium concentrations.

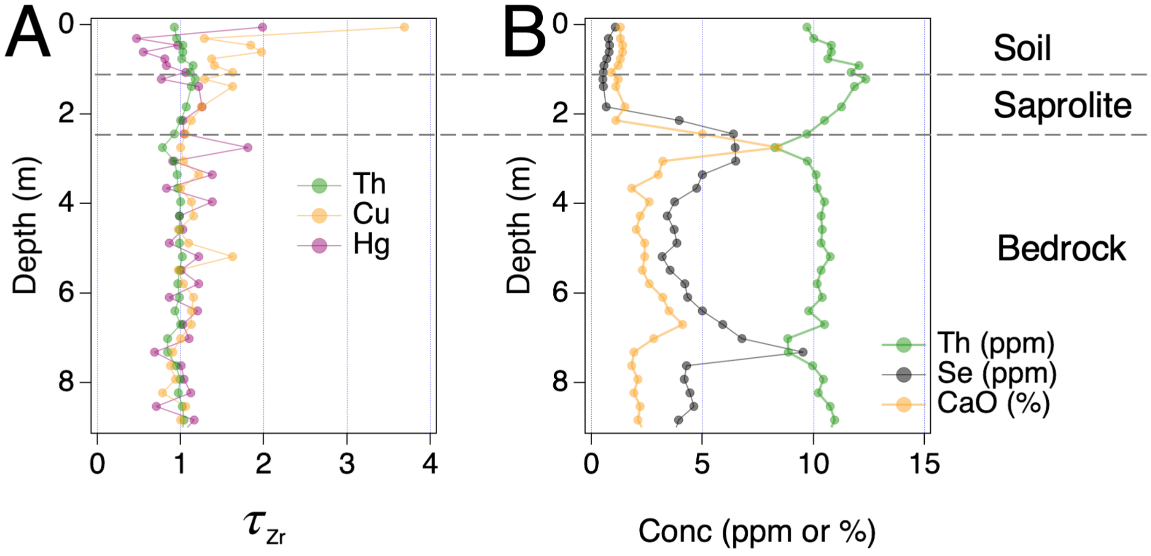

Figure S7 Vertical elemental profiles of selected elements in soil, saprolite and bedrock from the PLM 6 location close to the Pump House at East River. (**A**) Profiles of thorium, copper and mercury normalized to their average values at 10 m show accumulation soil surface accumulation of Cu and Hg but not Th. (**B**) Comparison of thorium, selenium and calcium as CaO. Redox-sensitive selenium accumulates at approximately 2.5 m, the depth of lowest groundwater depth and onset of the permanently anaerobic subsurface. Thorium is depleted in regions of Se accumulation, suggesting preferential loss in high-flow anaerobic zones.

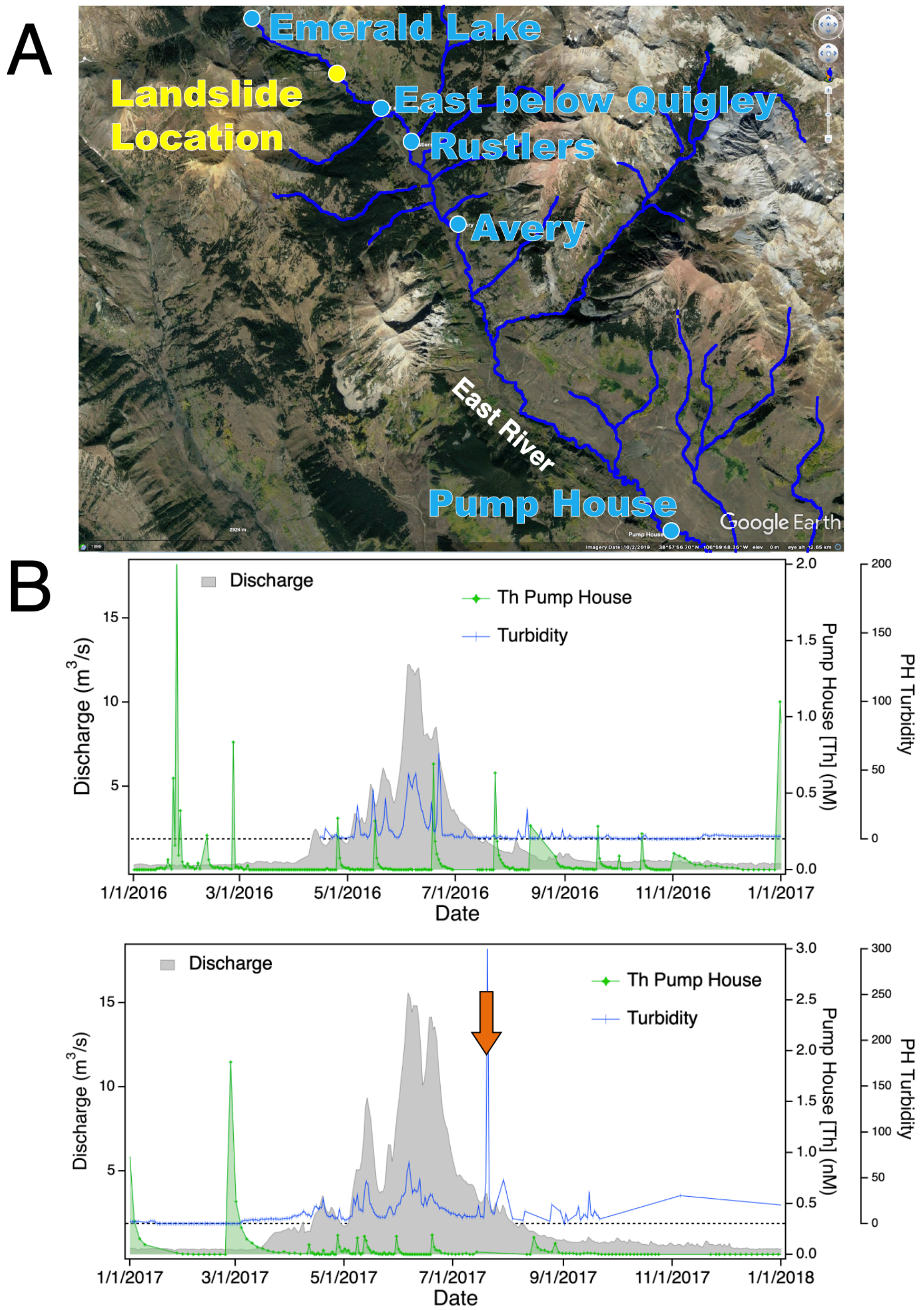

Figure S8 A landslide transported surface material into the East River some point between July 19 – 27th 2017. (**A**) Approximate landslide location. Satellite image was obtained from Google Earth Pro. (**B**) East river stream chemistry and turbidity. A power failure prevented stream water chemistry sampling between July 13th to August 10th. Continuous turbidity monitoring captured a large turbidity excursion (arrow) that was likely caused by the landslide.

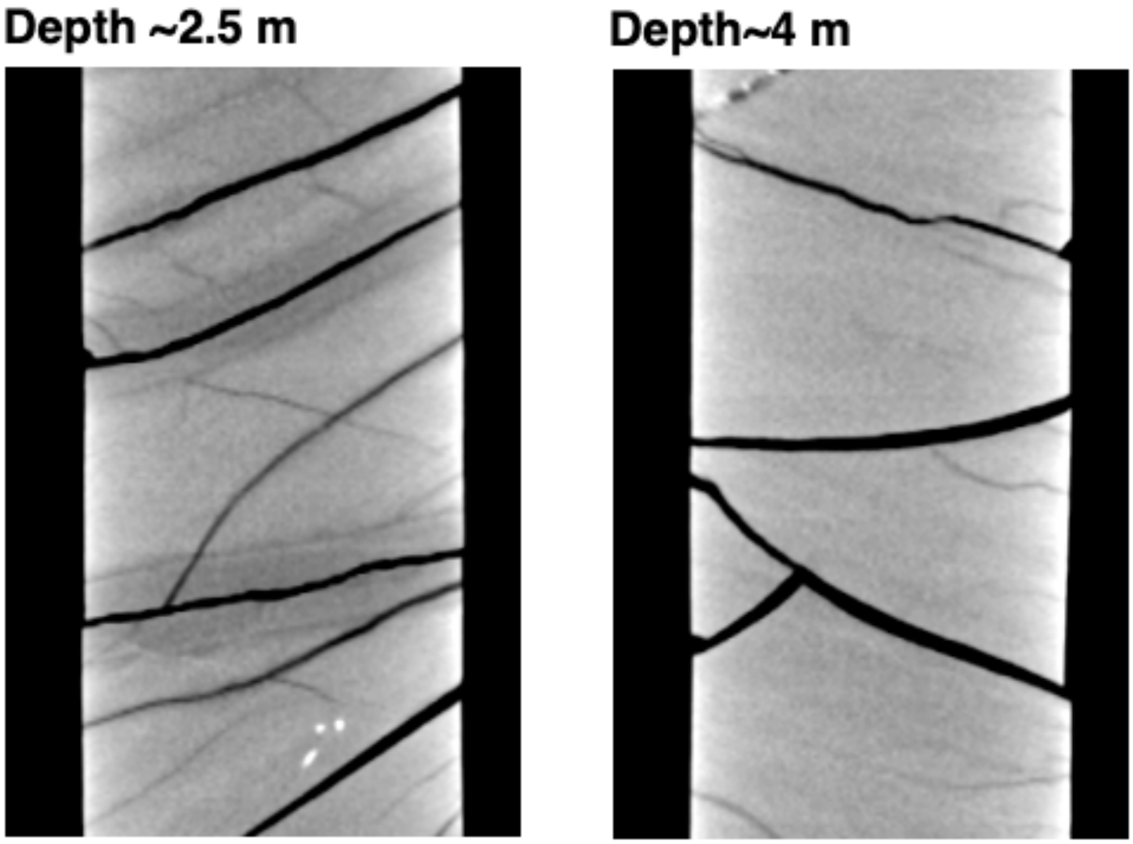

Figure S9 X-ray computed tomography (XCT) imaging of fractures from two depths in a 4-inch diameter core from the PLM 6 location in the East River. The fractures at a depth of 2.5 m are close to the water table and show darker (lower density) regions close to the fracture surfaces caused by chemical weathering and the release of elements into groundwater. The fractures at lower depths show no comparable density profiles and are unweathered.

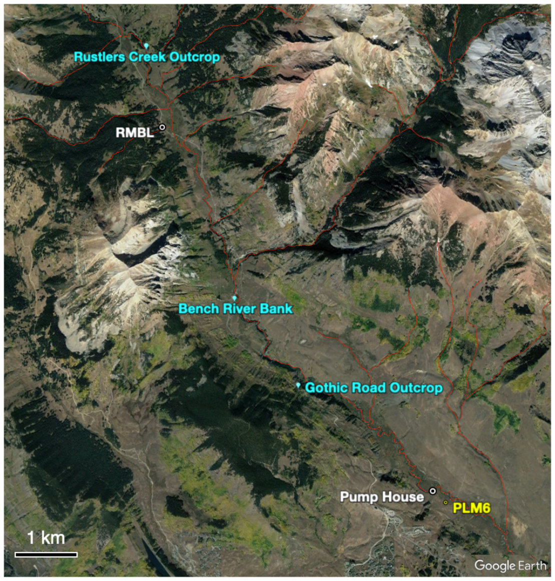

Figure S10 Locations of drill cores used for laboratory leaching test. Satellite image was obtained from Google Earth Pro.

	Thorium (ppb)				Color
	Anoxic		Oxic		< 1.0 ppb
**Location**	**pH 4**	**pH 7**	**pH 4**	**pH 7**	
Analytical Control	0.12	0.09	0.01	0.01	
Rustlers Creek Outcrop	0.57	0.20	0.17	0.04	
Bench River Bank	0.73	0.63	0.09	0.09	
Gothic Road Outcrop	0.67	0.26	0.05	0.11	
Pump House PLM 6 Drill Core	1.00	0.43	0.13	0.45	
Pump House PLM 6 Soil			0.47	0.43	>0.01 ppb

	Arsenic (ppb)				Color
	Anoxic		Oxic		< 11 ppb
**Location**	**pH 4**	**pH 7**	**pH 4**	**pH 7**	
Analytical Control	0.54	0.00	0.16	0.02	
Rustlers Creek Outcrop	1.48	1.07	1.69	1.05	
Bench River Bank	1.01	1.07	1.19	1.10	
Gothic Road Outcrop	1.11	1.82	1.03	0.68	
Pump House PLM 6 Drill Core	3.87	10.35	3.39	9.90	
Pump House PLM 6 Soil			5.92	5.86	>0.01 ppb

Table S2 Thorium and arsenic leaching from Mancos Shale

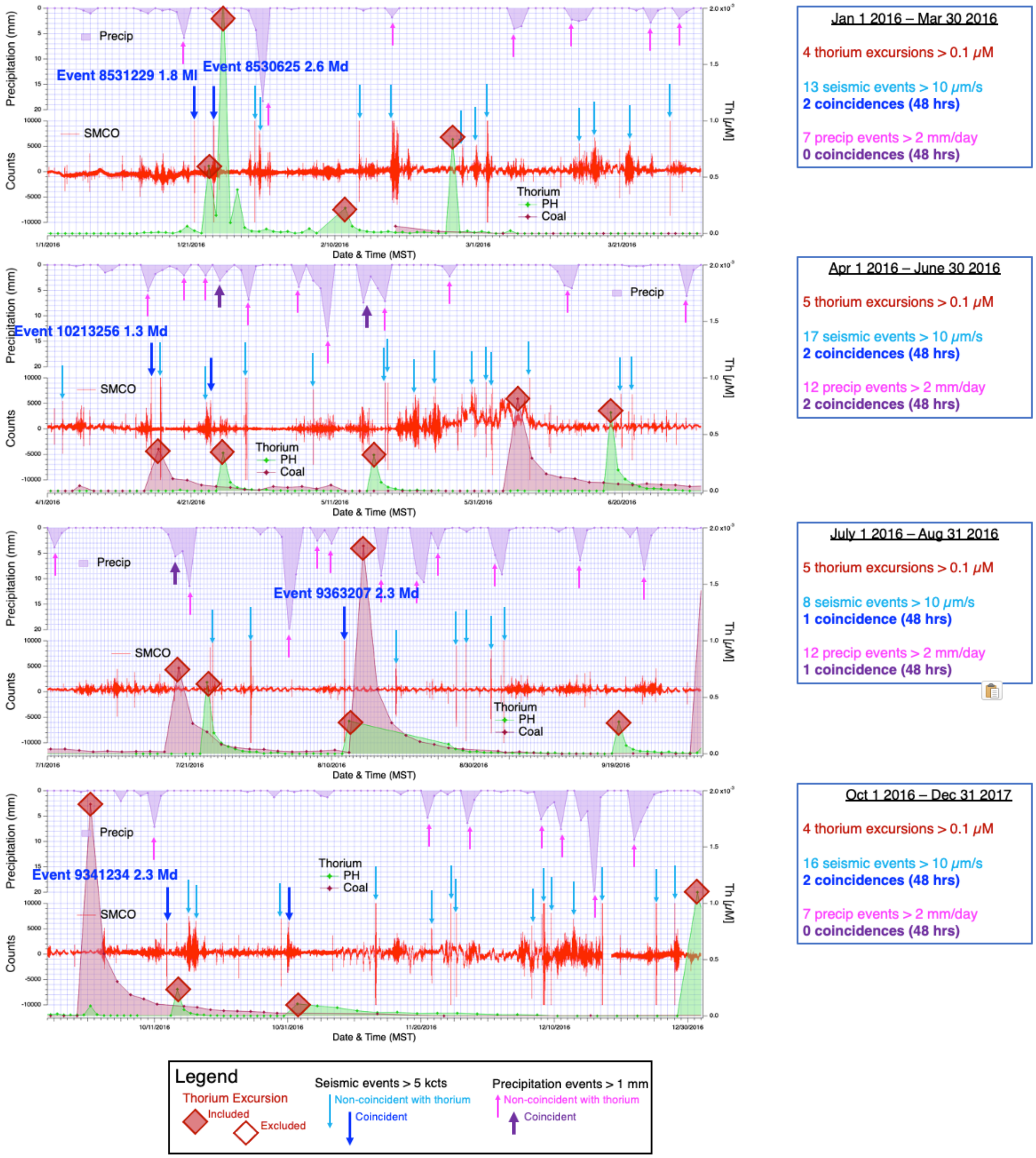

Fig. S11 (partial) 2016 January–December thorium concentrations in East River and Coal Creek compared with Snowmass, CO, seismicity data and KCOMKRET2 precipitation data. Data are plotted in 3-month intervals. See next page for full figure caption.

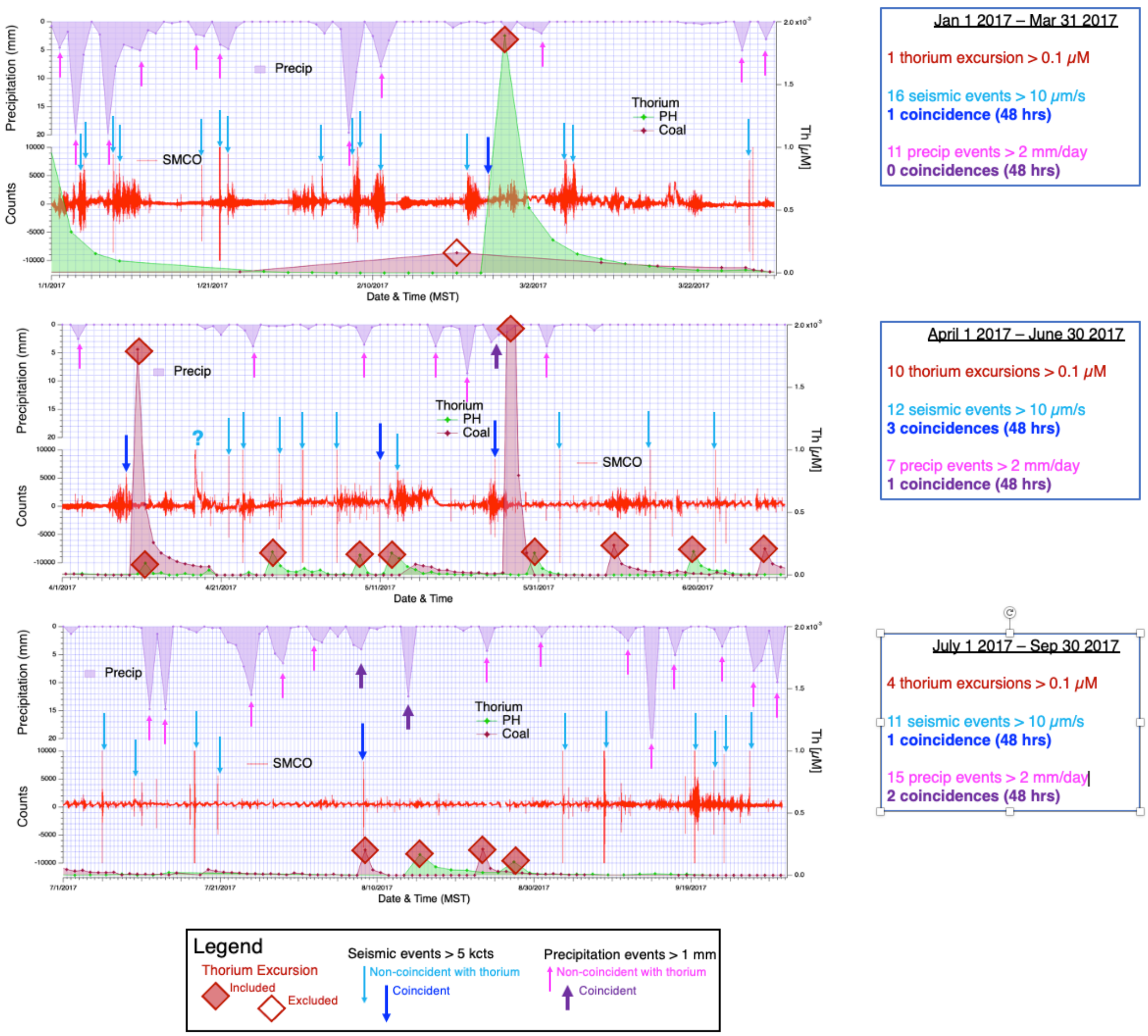

Fig. S11 (continued) 2017 January–September thorium concentrations in East River and Coal Creek in calendar compared with Snowmass, CO, seismicity data and KCOMKRET2 precipitation data. Data are plotted in 3-month intervals. As shown in the legend, full and empty diamond markers label the thorium excursions detected at the East River Pump House (PH) or Coal Creek sampling locations that were included in the statistical analysis. Seismic or precipitation events that preceded Th excursions by up to 48 hours were labeled as “coincident” and indicated with the thicker arrows.

Date	Site	[Th] μM	ISCO (cts)	Hours	Event	Magnitude	Latitude	Longitude	Location
1/23/2016	PH	0.60	80,000	48	8531229	1.8 Ml	34.049	-116.385	CA
1/25/2016	PH	2.2	25,000	24	8530625	2.6 Md	35.710	-121.052	CA
2/12/2016	CC	0.28	—	—					
2/26/2016	PH	0.84	—	—					
4/5/2016	CC	0.05	—	—					
4/16/2016	CC	0.38	18,900	24	10213256	1.3 Md	35.996	-120.554	CA
4/25/2016	PH	0.33	5,400	48	none				
5/16/2016	PH	0.31	4,500	48					
6/5/2016	CC	0.81	—	—					
6/18/2016	PH	0.69	—	—					
7/19/2016	CC	0.76	—	—					
7/23/2016	PH	0.63	900	24					
8/12/2016	PH	0.29	14,200	24	9363207	2.3 Md	37.632	-118.939	CA/NV
8/13/2016	CC	1.80	14,200	48	9363207	2.3 Md	37.632	-118.939	CA/NV
10/1/2016	PH	0.09	3,900	48					
10/1/2016	CC	1.90	3,900	48					
10/14/2016	PH	0.24	8,300	48	9341234	2.1 Md	37.634	-118.877	CA/NV
11/1/2016	PH	0.11	5,050	24					
12/31/2016	PH	1.10	—	—					

Table S3 Thorium excursions from 2016 preceded by seismicity.

Event Type	N_A	N_B	K_{precip}	$K_{seismic}$	P_{anal}	P_{MC}
Thorium	—	33	—	—	—	
Precipitation	71	—	6	—	0.670	0.657
Seismicity	93	—	—	12	0.107	0.115

Table S4 Event coincidence analysis for the example given in Fig. S11. Comparison of analytical and Monte Carlo calculations of the *p*-values testing the null hypothesis that *K* observations of precipitation or seismicity preceded thorium excursions.

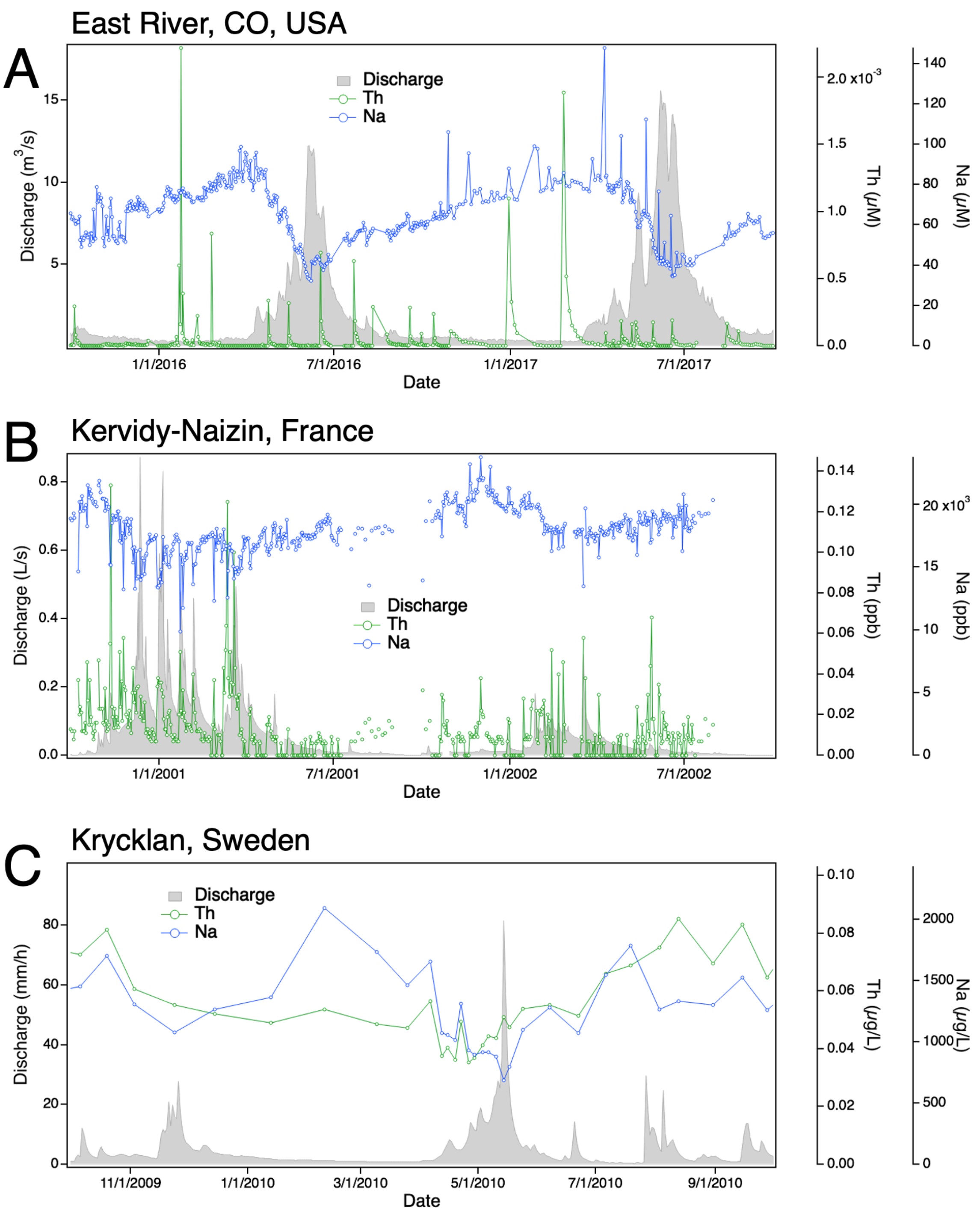

Figure S12 Comparison of East River thorium data with other field sites. (**A**) East River, CO, data from this manuscript (**B**) Kervidy-Naizin, France. (**C**) Kycklan, Sweden.

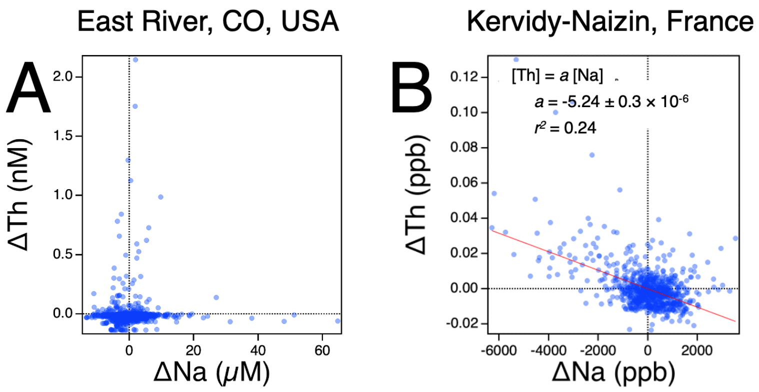

Figure S13 Detrended correlations between thorium and sodium at two field sites. (**A**) East River, CO, data from this manuscript (**B**) Kervidy-Naizin, France.
